# Is agricultural engagement associated with lower incidence or prevalence of cardiovascular diseases and cardiovascular disease risk factors? A systematic review of observational studies from low- and middle-income countries

**DOI:** 10.1371/journal.pone.0230744

**Published:** 2020-03-31

**Authors:** Tina B. Sørensen, Mika Matsuzaki, John Gregson, Sanjay Kinra, Suneetha Kadiyala, Bhavani Shankar, Alan D. Dangour

**Affiliations:** 1 Department of Population Health, Faculty of Epidemiology and Population Health, London School of Hygiene & Tropical Medicine, London, United Kingdom; 2 Department of Non-communicable Disease Epidemiology, Faculty of Epidemiology and Population Health, London School of Hygiene & Tropical Medicine, London, United Kingdom; 3 London Centre for Integrative Research in Agriculture and Health (LCIRAH), London School of Hygiene & Tropical Medicine, London, United Kingdom; 4 Centre for Development, Environment and Policy, School of Oriental and African Studies, London, United Kingdom; Purdue University, UNITED STATES

## Abstract

Non-communicable diseases, such as cardiovascular diseases (CVDs), diabetes and cancer account for more than half of the global disease burden, and 75% of related deaths occur in low- and middle-income countries (LMICs). Despite large regional variations in CVD incidence and prevalence, CVDs remain the leading causes of death worldwide. With urbanisation, developing nations are undergoing unprecedented labour-force transitions out of agriculture and into types of non-agricultural employment, mainly in the industry and service sectors. There are few studies on the effect of these transitions on CVDs and CVD risk factors in LMICs. We systematically searched MEDLINE, PubMed, EMBASE and the Cochrane Library from January 1950 to January 2017 to assess the association of engaging in agriculture compared to types of non-agricultural employment (e.g. services and manufacturing) with CVD incidence, prevalence and risk factors. Studies were included if they: included participants who engaged in agriculture and participants who did not engage in agriculture; measured atherosclerotic CVDs or their modifiable risk factors; and involved adults from LMICs. We assessed the quality of evidence in seven domains of each study. Prevalence ratios with 95% confidence intervals were calculated and compared in forest plots across studies. Study heterogeneity did not permit formal meta-analyses with pooled results. There was a lack of publications on the primary outcomes, atherosclerotic CVDs (n = 2). Limited evidence of varying consistency from 13 studies in five countries reported that compared with non-agricultural workers, mainly living in urban areas, rural agriculture workers had a lower prevalence of hypertension, overweight and obesity; and a higher prevalence of underweight and smoking. High quality evidence is lacking on the associations of engaging in and transitioning out of agriculture with atherosclerotic CVDs and their modifiable risk factors in LMICs. There is a need for interdisciplinary longitudinal studies to understand associations of types of employment and labour-force transitions with CVD burdens in LMICs.

## Introduction

Non-communicable diseases (NCDs), such as cardiovascular diseases (CVDs), diabetes and cancers are the leading causes of death and disability worldwide, accounting for more than half of the global disease burden.[1] Almost 75% of NCD-related deaths occur in low- and middle-income countries (LMIC),[2] often among working-age adults as young as 40 years.[3] Although disease patterns vary across world regions, CVDs remain the leading causes of death throughout.[4] Most CVDs develop from atherosclerosis (the hardening and narrowing of major blood vessels).[5] As such, atherosclerotic CVDs are largely preventable by addressing risk factors including unhealthy diets, physical inactivity, harmful use of alcohol, tobacco use, hypertension, overweight and obesity, diabetes and dyslipidaemia.[5–7] Urbanisation is demanding still more non-agricultural labour, and LMICs have undergone unprecedented labour-force transitions out of agriculture and into the industry and service sectors (Table 1).[8, 9] Labour-force transitions out of agriculture have been particularly steep in middle-income countries such as China, however, large low-income countries, such as India, are quickly catching up.

**Table 1:** Percent employment in agriculture, services and industry of total employment in low-and middle-income countries.

	% of total employment		
	1991	2004	2018
**Low-income countries**			
Agriculture	71	70	63
Services	20	21	26
Industry	9	9	11
**Low- and middle-income countries**			
Agriculture	53	46	34
Services	27	34	43
Industry	19	20	23
**Upper middle-income countries**			
Agriculture	48	37	22
Services	29	38	52
Industry	24	25	27
**India**			
Agriculture	63	57	44
Services	22	25	31
Industry	15	18	25

Source World Bank Group[10]

Type of employment is an important social determinant of health.[11] Types of employment contribute significantly to shaping the conditions of daily life that strongly associate with major immediate CVD risk factors such as hypertension, obesity, diabetes and dyslipidaemia.[11–13] For example, the type, amount and stability of labour and income influence people’s ability to acquire diverse and nutritious foods (through access to own-produce or purchase), assets and health-services.[13, 14] The type and duration of labour influence physical activity, nutritional needs, time available for food preparation and levels of exposure to biological and chemical hazards.[13, 15, 16] The environment in which people live and work influence the availability, access and affordability of commodities that may have beneficial or harmful effects on cardiovascular health, such as fruits and vegetables, highly processed energy-dense foods, tobacco and alcohol.[13, 16–18]

Agriculture has the potential to benefit nutrition and cardiovascular health, through the increased production and availability of nutritious foods and higher levels of physical activity that is associated with agricultural labour.[14, 15] Engaging in agriculture is also associated with cardiovascular health risks such as prolonged exposure to disease vectors, food borne diseases and toxic pesticides.[13] Most systematic reviews on the links between agriculture, nutrition and cardiovascular health synthesise evidence from studies that introduce, improve or intensify agriculture. In light of the expected continued labour-force transitions away from agriculture in LMICs, this paper aims to address two additional questions (i) is engaging in agriculture compared to types of non-agricultural employment (e.g. services and manufacturing) associated with lower levels of CVD incidence, prevalence and risk factors? (ii) Is the process of transitioning out of agriculture and into types of non-agricultural employment associated with higher levels of CVD incidence, prevalence and risk factors? Our initial systematic search returned only one eligible study pertaining to question (ii) and we therefore set out systematically to review the published evidence of the associations of engaging in agriculture compared to (any) types of non-agricultural employment with CVD incidence, prevalence or CVD risk factors in LMICs. We hypothesised that people who engage in agriculture have lower levels of CVDs and associated risk factors (not considering chemical, ambient and noise pollutants) than individuals who engage in other types of labour, particularly types of sedentary work. A review of the evidence might identify types of higher-risk employment, and with that, provide guidance to categorising employment in future longitudinal studies of employment transitions out of agriculture.

## Methods

This systematic review asked the following questions (i) is engaging in agriculture compared to types of non-agricultural employment (e.g. services and manufacturing) associated with lower levels of CVD incidence, prevalence and risk factors?; and (ii) is the process of transitioning out of agriculture and into types of non-agricultural employment associated with higher levels of CVD incidence, prevalence and risk factors? The review is reported according to the Preferred Reporting Items for Systematic Reviews and Meta-Analyses (PRISMA) [19] (see S1 Checklist) and the Guidelines for Meta-Analyses and Systematic Reviews of Observational Studies.[20] The protocol was published in advance (ID=CRD42015025488).[21] Eligibility criteria were defined in relation to PICOS (participants, interventions, comparisons, outcomes, and study design) as recommended by PRISMA.

### Eligibility criteria

#### Population

We included studies that reported on individuals i) from at least one LMIC as defined by the World Bank at the time of the study[22] and ii) aged 15 years and above or described as ‘adults’, ‘men’ or ‘women’.

#### Interventions, exposures and comparators

Following the Food and Agriculture Organization’s (FAO) definition, we defined ‘agriculture’ as horticulture and agro-forestry (e.g. preparing the soil, planting, fertilising, weeding, watering or harvesting food or other crops) as well as animal husbandry (e.g. rearing, feeding, breading and caring for animals used for food, wool/fur or economic purposes), beekeeping, aquaculture, fishing and hunting.

Studies were eligible for inclusion if one group of participants reported to engage in agriculture on their own or someone else’s land, either as a primary occupation or by predominantly depending on agriculture for their livelihood. Studies were included if they had at least one comparator group of participants who reported to not engage in agriculture as defined above. To reduce contamination of comparator groups, we excluded studies that sourced comparator groups from ‘agricultural communities’ or similar without specifying if participants engaged in agriculture.

#### Outcomes

Studies were included if they measured one or more atherosclerotic CVDs (primary outcomes) or related modifiable risk factors (secondary outcomes) (Box 1).[6, 23–25] When multiple publications analysed data from one study, we included results for all unique outcomes. When multiple publications presented overlapping analyses from the same study, we included results from the most comprehensive analysis based on methods and sample size.

**Box 1. Atherosclerotic cardiovascular diseases (primary outcomes) and their modifiable risk factors (secondary outcomes)**

**Atherosclerotic cardiovascular diseases and events**
Ischaemic heart disease or coronary artery/heart disease, for example heart attack
Cerebrovascular disease, for example stroke
Peripheral vascular disease
Deep vein thrombosis
Pulmonary embolism
Unspecified cardiovascular disease (CVD)
Specified or unspecified CVD mortality

**Diet**
Saturated fat, trans fat, cholesterol, carbohydrate (including sugar), fibre, antioxidants: vitamin C, E, Ubiquinone (coenzyme Q10), bioflavonoids, selenium, folate, vitamin B6, vitamin B12, potassium, fruits and vegetables, whole grain cereals, unsalted nuts, fish, salt/sodium

**Physical activity**
(Low) physical activity

**Tobacco and alcohol**
Any or harmful alcohol consumption
Any tobacco use

**Metabolic cardiovascular disease risk factors**
Body mass index
Overweight and obesity
Underweight
Systolic and diastolic blood pressure
Hypertension
(High) total cholesterol
(Low) high-density lipoprotein
(High) low-density lipoprotein
Total cholesterol:high-density lipoprotein ratio
(High) triglycerides
Fasting glucose
Impaired fasting glucose
Diabetes Mellitus Type II
Augmentation index
Carotid intima-media thickness
Homocysteine
High sensitivity C—reactive protein
(High) serum apolipoprotein B
(Low) serum apolipoprotein A-I

**Composite measures**
CVD risk score, e.g. the Framingham 10-year coronary heart disease risk score
Homeostatic model assessment
Dyslipidaemia
Metabolic syndrome
CVD–cardiovascular disease
Sources[6, 23–25]

#### Study design

We included comparative studies of any design and duration. No restriction was placed on sample size in the initial review phase. However, because of their limited generalisability and power, case studies and studies with small sample sizes (typically between 30 and 50 participants) were excluded. The characteristics of excluded studies can be found in S2 Table in the online information.

#### Data

We included studies that, as a minimum, reported crude estimates of associations, e.g. prevalence ratios (PRs) and 95% confidence intervals (CIs), or enough information to calculate them. Studies that in addition to crude estimates provided adjusted estimates (of any type) were eligible if they did not also adjust for mediators (factors on the causal pathway between employment and CVDs or risk factors) in non-mediation analyses. We restricted analyses to risk factors that were reported in four or more studies and for which clear descriptions of measurement methods and outcome categorisations were provided. In addition to the main analysis, we summarised studies on primary outcomes, atherosclerotic CVDs, if they met eligibility criteria other than that relating to ‘four or more available studies’.

### Information sources and search strategy

Our search was conducted in January 2017 and searched databases dating back to January 1950. We systematically searched the electronic databases MEDLINE, PubMed, EMBASE and the Cochrane Library by using key words and Medical Subject Headings delimiting ‘agriculture’ and ‘CVD’ or ‘CVD risk factors’ (S1 Table). The search was limited to human subjects and texts in English, Danish, Norwegian, Swedish, German and Spanish. We manually searched bibliographies of included primary publications, relevant reviews and supplementary grey literature (the latter identified from Google and Google Scholar searches). We included only primary peer-reviewed literature in the final review.

### Selection process and data extraction

We imported and managed citations in Endnote X7. Two investigators (TBS and MM) individually screened all titles and abstracts against the eligibility criteria and resolved any disagreement in study selection by discussion. One reviewer (TBS) used data extraction forms that were designed for the review to extract data on PICOS and data were imported into Excel 2016. A second reviewer (MM) double-checked the extracted data against the original publications.

### Quality of the evidence

We adapted ‘A Cochrane Risk of Bias Assessment Tool: for Non-Randomized Studies of Interventions (ACROBAT-NRSI)[26] to assess the quality of evidence. The assessment covered seven risk of bias domains: confounding, selection of study participants, measurement of exposures/interventions, departures from intended interventions, missing data, measurement of outcomes and selection of the reported results. We rated the quality of evidence within each domain and overall at the study level as ‘well covered’, ‘adequately addressed’, ‘poorly addressed’, ‘not applicable’, ‘not described adequately to classify’ or ‘not described’.

### Compliance with ethical standards

Ethical approval for the current systematic review was obtained from the London School of Hygiene and Tropical Medicine, London, United Kingdom. Informed consent was obtained from all participants in the original publications, from which the data for the current review were extracted.

### Analysis

Where outcome units differed between studies, we converted results to a common unit using biomedical research conversion tables.[27] Some studies described one risk factor with multiple estimates, e.g. mean body mass index (BMI) and prevalence of overweight and obesity. The type of information reported by most studies were included in the analysis, resulting in the exclusion of continuous data. Characteristics of studies that were excluded from the analysis as a result of these restrictions are available in S2 Table in the online information.

We used formulas suggested by Sterne (Eq (1))[28] to calculate PRs with 95% CIs and produce forest plots that graphically describe patterns of outcomes by livelihood or occupation groups across studies. Adjusted PRs (95% CIs) could not be calculated because we did not have access to raw datasets. Narrative analyses of adjusted estimates were presented separately. It was not appropriate to perform formal meta-analyses or generate funnel plots because of the substantial heterogeneity of measurement methods, categorisation of exposures and outcomes, study settings and populations of the included studies. All analyses were performed in Stata 14.

Logprevalenceratio=log([exposedcases/totalexposed]/[unexposedcases/totalunexposed])

Standarderroroflogprevalenceratio=√(1/exposedcases+1/unexposedcases−1/totalexposed−1/totalunexposed) (1)

## Results

**Fig 1:**
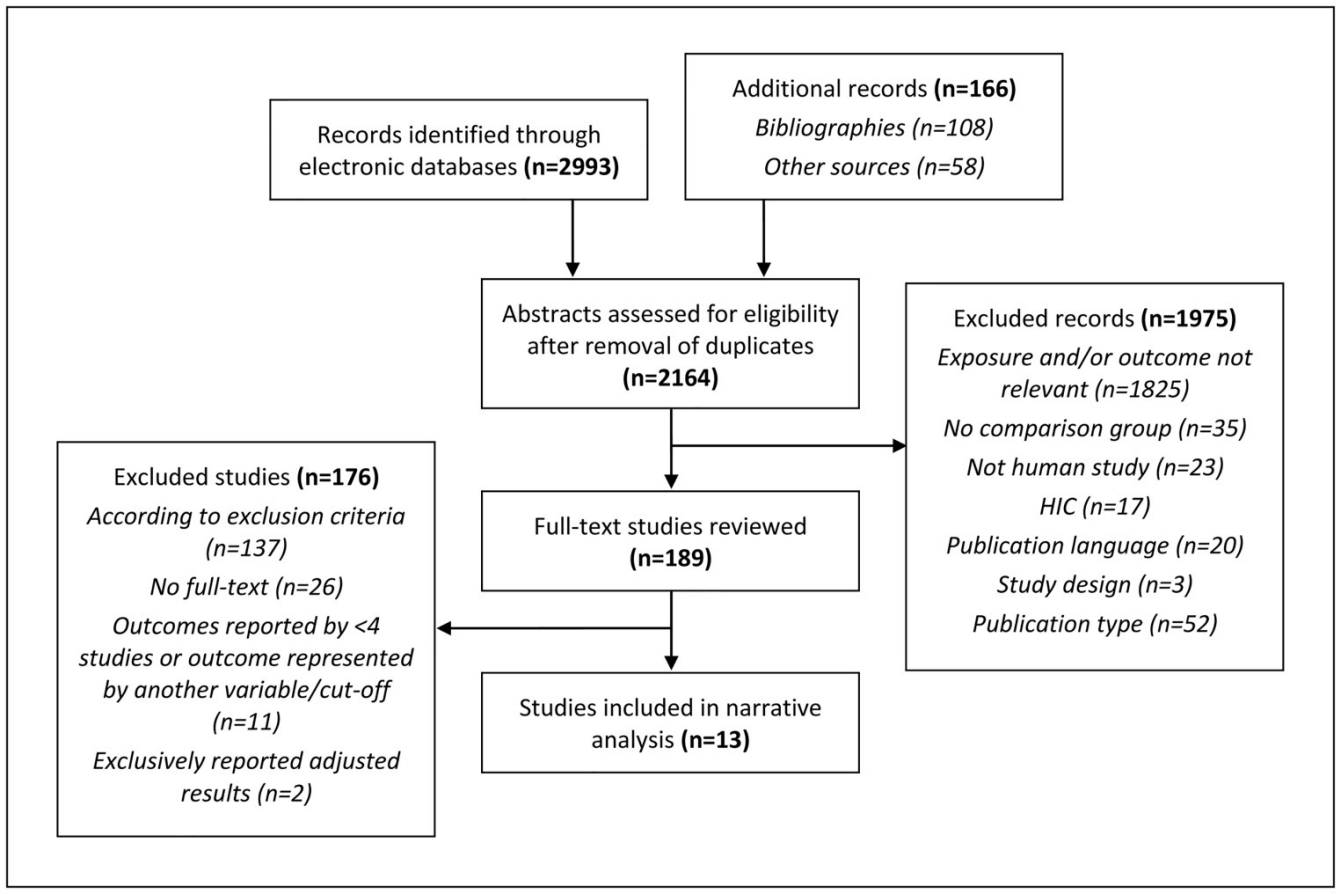
Flow diagram of the review process. CVD–cardiovascular disease, HIC–high-income country, n–number of studies.

Our search yielded 3159 records, including 166 studies identified through other sources, such as manually searching bibliographies and contacting authors (Fig 1). After removing duplicates (n = 995) we screened 2164 titles and abstracts and reviewed 189 full-text publications against inclusion/exclusion criteria. We included 13 publications that reported data from 12 unique studies. Only one eligible study provided appropriately adjusted estimates (i.e. not including mediators in non-mediation analysis). Adjusted results on hypertension from this study is presented separately. A summary of identified studies on primary outcomes, atherosclerotic CVDs (n = 2), which did not meet inclusion/exclusion criteria for the main analysis, is further provided.

### Study characteristics

Characteristics of included studies are provided in Table 2 (see S3 Table for further details). We included one longitudinal[29] and 12 cross-sectional studies. The longitudinal study followed up participants 10 years from baseline. The cross-sectional studies comprised eight analyses of primary data[30–36]; two secondary analyses of existing cross-sectional data[37, 38]; and three cross-sectional analyses of longitudinal data,[39–41] of which two used data from different time points of one study.[40, 41] Study settings and characteristics differed substantially between studies. Most studies were from Asia (India[31, 33, 35–39] and China[29, 40, 41]); one was from Latin America (Guatemala[32]) and two were from Sub-Saharan Africa (Ghana[30] and Nigeria[34]). Eight studies analysed data from rural populations and five studies additionally included urban participants (including migrants). Sample sizes ranged from 195 to 77,220 participants. Twelve studies reported the age range of participants; eight included younger adults aged from 15 years,[38, 42] 18 years,[30, 36, 39] and 20 years.[34, 35, 41] The maximum age of participants varied from 49 to 99 years. One study did not report the age range, however, described participants as adults.[32]

**Table 2:** Characteristics of included studies from five low- and middle-income countries (n = 13).

Author and year	Population and site	Study design	Outcomes	Case definition	Exposure and comparators	n	Age, mean (SD)	Age range in years
Addo et al. 2006	Ghana, four rural farming communities	Cross-sectional	Hypertension	≥140/90 mmHg	Farmer	107	42.4 (18.6)	18, 99
					Trader	152		
					Other	103		
Arlappa et al. 2009	India, rural areas in nine states	Cross-sectional	Underweight	BMI <18.5 kg/m^2^	Agriculture	399		60, 70+
					Non-agriculture	1,170		
Asgary et al. 2013	Jamkhed, India, six rural villages	Cross-sectional	Hypertension	≥140/90 mmHg	Farmer	112		40, 85
					Housekeeper	100		
Balagopal et al. 2012	Gujarat, India, rural community	Cross-sectional	Hypertension; underweight, overweight, obese; tobacco	SBP ≥140 mmHg; BMI <18.5, 23–24.99, ≥25 kg/m^2^	Agrarian (low socio-economic status)	764	43.4 (15.9)	18+
					Business (high socio-economic status)	874	40.2 (15.7)	
Gregory et al. 2007	Guatemala, people born in four rural villages	Cross-sectional	Hypertension; overweight, obese; smoking	≥130/85 mmHg; BMI ≥25, ≥30 kg/m^2^	Rural agriculture	88	31.7 (4.4)	
					Rural non-agriculture	153	31.4 (4.2)	
					Urban	119	33.6 (4.3)	
Hazarika et al. 2004	Assam, India, 25 rural villages	Cross-sectional	Hypertension	≥140/90 mmHg	Service			≥30
					Business			
					Cultivator			
					Daily wager			
					Unemployed			
					Others			
					Total	3,180		
He et al. 1991	Sichuan province, China, mountains, city and county seats	Cross-sectional	Age standardised hypertension I and II; smoking	140-159/90-94, ≥160/95 mmHg	Farmer	8,241	31.4	15, 89
					Migrant	2,575	33.1	
					Urban	3,689	33.9	
Norboo et al. 2015	Jammu and Kashmir, India, rural and urban areas	Cross-sectional	Hypertension; overweight	≥140/90 mmHg, BMI ≥25 kg/m^2^	Farmer	1,247		20, 94
					Nomad	220		
					Sedentary worker	549		
					Other, including:	784		
					*Housewife*	*325*		
					*Manual labourer*	*63*		
					*Monk*	*157*		
					*No job*	*138*		
					*Retired sedentary*	*101*		
					Total	*2*,*800*	53.8 (15.0)	
Olugbile & Oyemade 1982	Nigeria, two rural areas in different states	Cross-sectional	Hypertension	≥140/90 mmHg	Agriculture company	112		20, 59
					Factory worker	136		
Subasinghe et al. 2014	Andhra Pradesh, India, 12 rural villages	Cross-sectional	Underweight	BMI <18 kg/m^2^	Non-government, government	376		18, 55+
					Self-employed	165		
					Farming and livestock	326		
					Homemaker	209		
					Unemployed, student, retired	93		
Subramanian & Davey Smith 2006	India, rural and urban areas in 26 states	Cross-sectional	Underweight, overweight, obese	BMI <16; 16–16.9, 17–18.49, <18.5; 23–24.9, 25–29.9; ≥30 kg/m^2^	Not working	48,160		15, 49
					Non-manual	4,433		
					Agricultural	17,758		
					Manual	6,869		
Wang et al. 2010	South-western China, mountains, city and county seats	Cross-sectional	Hypertension; overweight/obesity; smoking	≥130/85 mmHg; BMI ≥24 kg/m^2^	Farmer	1,535	39.6	≥20
					Migrant	1,306	38.8	
					Urban	2,130	44.3	
Zhou et al. 2003	Beijing, Northern China, and Guangzhou, Southern China, rural areas near big cities	Cohort	Smoking		Agriculture 1983–84	326		35, 54 (at baseline)
					Remained in agriculture 1993–94			
					Agriculture 1983–84	102		
					Shifted out of agriculture 1993–94			
					Factory work 1983–84	135		
					Remained in factory work 1993–94			
					Office work 1983–84	70		
					Remained in office work 1993–94			

BMI—body mass index; HH—household(s); kg–kilograms; m—metre(s); mmHg—millimetre mercury; n–sample size; SBP—systolic blood pressure

### Quality of the evidence

We identified substantial methodological shortcomings in all included studies (S4 Table). A particular concern was that none of the included studies described the measurements of exposure and comparators or outcomes in adequate detail to rate the quality of evidence relating to these domains. Studies rarely defined agriculture, but described participants in broad terms, such as farmers or agriculturalists. Two studies described participants’ agricultural practices in more detail, such as whether they farmed their own, leased or someone else’s land. The included 13 studies explored associations of engaging in agriculture with secondary outcomes, modifiable risk factors for atherosclerotic CVDs. Study outcomes were measured and categorised in various ways across the studies. None of the studies addressed blinding of outcome assessors although all assessed outcomes might be subjective and therefore open to bias from either assessors or participants.

### Primary outcomes

Two studies reported on primary outcomes (S2 Table). One study from India classified participants as having coronary heart disease (CHD) if angina or infarction and CHD had previously been diagnosed; affirmative response was given to the Rose questionnaire; or changes were observed in electrocardiograms according to the Minnesota code classification system. The prevalence of CHD did not differ between agricultural, business, professional, government and household workers (n = 3,148).[43] The second study, from Vietnam, combined mortality, identified by verbal autopsy, from undifferentiated CVDs, pulmonary heart disease, stroke and CHD during a two year period (n = 49,543 person-years).[44] The rate of CVD was almost six times higher among non-pension retired individuals than among farmers in a subsample of older participants (≥ 50 years, n = 15,193 person-years). The association more than halved when adjusting for age and gender.[44] Farmers did not differ from government employees and ‘others’.

### Secondary outcomes

#### Hypertension

**Fig 2:**
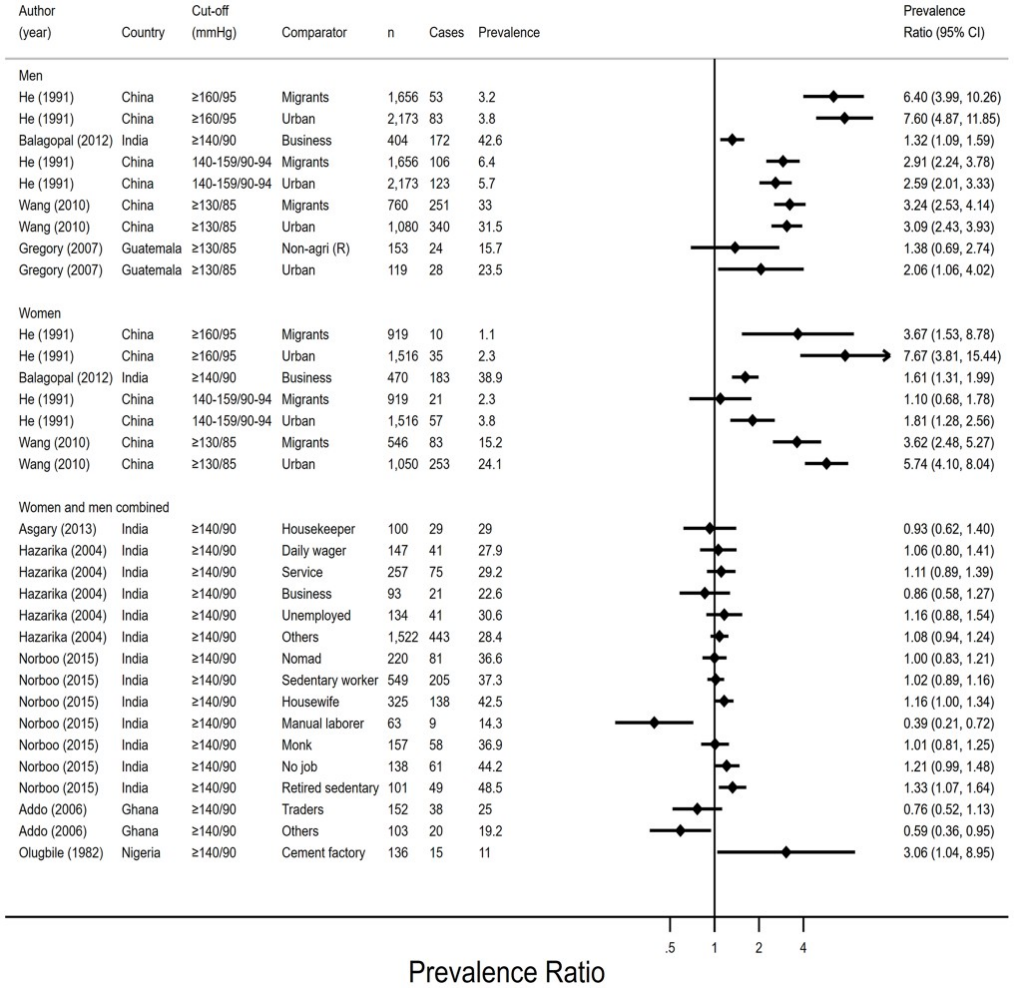
Prevalence ratios and 95% confidence intervals of hypertension by employment status (n = 9). Agri–agriculture, CI–confidence interval, n–sample size, PR–prevalence ratio, R–rural. Prevalence ratios were derived from comparing each non-agricultural group (coded 1) to the agricultural group (coded 0).

Nine studies reported sufficient data for calculating PRs (95% CIs) of hypertension in agricultural and non-agricultural groups. Seven studies were set in rural areas and two included rural and urban residents. The prevalence of hypertension varied considerably between compared groups and studies, from 0.3% among farmers in one study[40] to 48.5% among retired sedentary workers in another.[35] However, different cut-offs were used (range: 130/85mmHg, ≥ 160/95mmHg), which hampered direct comparisons of results (Fig 2). In three publications (from two studies), hypertension was less prevalent among farmers than migrants[40, 41] and urban workers,[32, 40, 41] with the exception of migrant women in one study.[40] The pattern was less clear in the studies that compared more specific occupation groups that combined men and women, who were predominantly living in rural areas. Farmers had a lower prevalence of hypertension than factory workers[34] and retired sedentary workers[35] in two studies. In two other studies, farmers had a higher prevalence of hypertension than manual labourers[35] and those with other employment than farming and trade.[30] Four studies did not observe differences in the prevalence of hypertension between agricultural and a range of non-agricultural groups comprising: non-farming rural workers,[32] housewives/housekeepers,[31, 35] daily wage earners,[33] service workers,[33] nomads,[35] sedentary workers,[35] monks,[35] the unemployed[33, 35] and a group of ‘others’ (who were not unemployed, business, service or daily wage workers).[33] Three comparisons of hypertension between agricultural workers and trade or business workers were contradictory: the prevalence was lower among agricultural workers than business workers in one study that analysed women and men separately[39] and similar between groups of agricultural and business/trade workers in analyses that combined women and men.[30, 33]

Hypertension remained considerably less common among farmers than migrants (urban people were excluded) after adjusting the odds ratios (ORs) for age in one study[40] (blood pressure ≥ 140-159/90-94 mmHg: OR^men^ 1.38 [95% CI 1.19, 1.59], OR^women^ 1.03 [95% CI 1.14, 2.93]; Blood pressure ≥ 160/95: OR^men^ 1.96 [95% CI 1.52, 2.52], OR^women^ 1.83 [95% CI 1.14, 2.93]). The adjusted OR was a slight attenuation among men and a slight increase among women compared to the crude ORs.

#### Overweight and obesity

**Fig 3:**
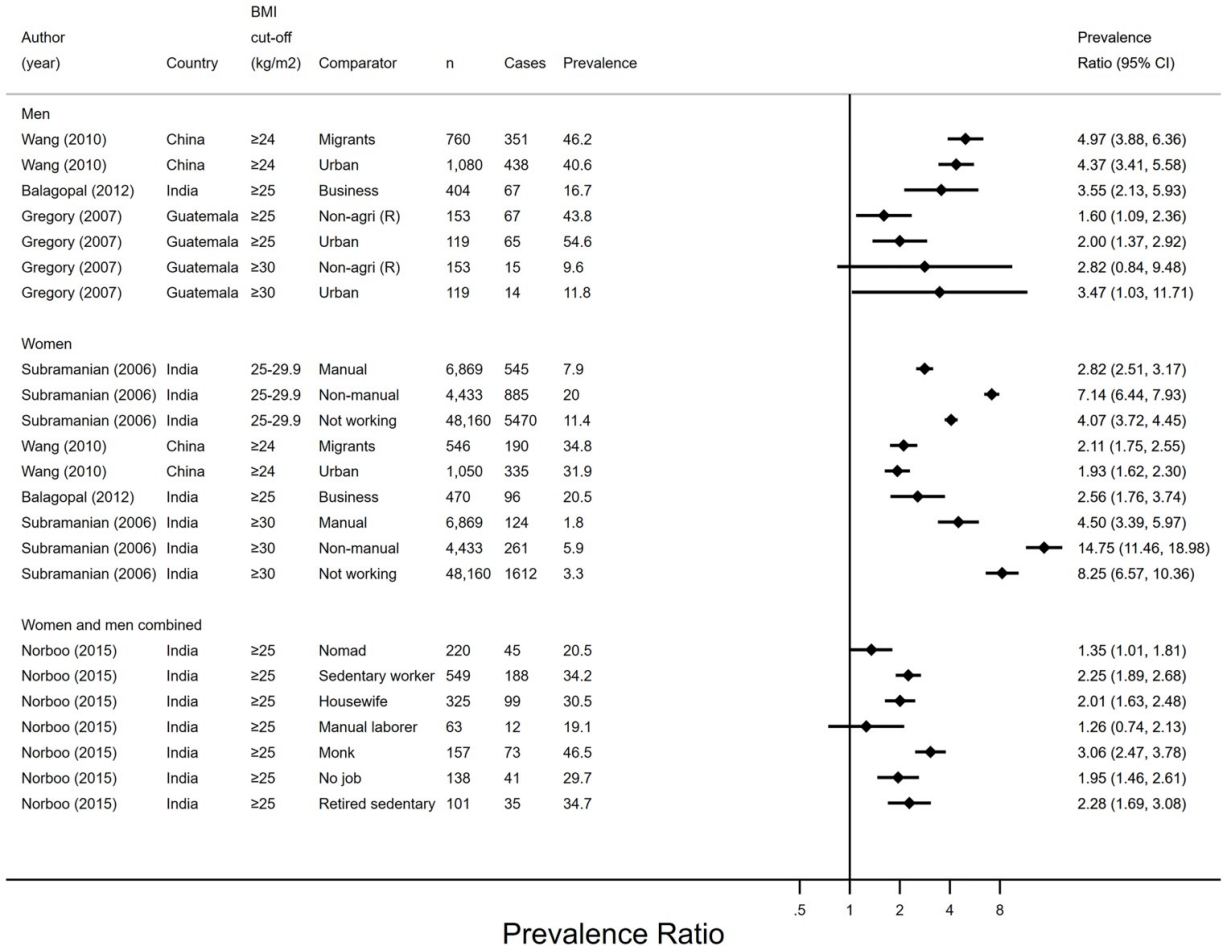
Prevalence ratios and 95% confidence intervals of overweight and obesity by employment status (n = 5). Agri–agriculture, BMI–body mass index, CI–confidence interval, n–sample size, PR–prevalence ratio; R–rural. Prevalence ratios were derived from comparing each non-agricultural group (coded 1) to the agricultural group (coded 0).

Five studies reported enough information to calculate PRs (95% CIs) for overweight and/or obesity. Studies predominantly combined overweight and obesity using international cut-offs (BMI ≥ 25 kg/m^2^) (Fig 3). Data from all five studies suggested that overweight and obesity (analysed separately and combined) were less prevalent among farmers than migrant,[41] urban,[32, 41] business,[39] non-manual,[38] sedentary[35] and retired sedentary workers[35]; nomads,[35] housewives,[35] monks,[35] and the unemployed.[35, 38] Overweight (BMI 25–29.9 kg/m^2^) and obesity (BMI ≥ 30 kg/m^2^) were less prevalent among women engaging in agriculture than manual labour in one study,[38] whereas overweight and obesity combined did not differ between agricultural and manual workers in another study combining men and women.[35] Overweight and obesity (BMI ≥ 25 kg/m^2^), but not obesity separately (BMI ≥ 30 kg/m^2^), was less prevalent among agricultural than non-agricultural rural men in one study.[32] However, large confidence intervals of the association suggested low precision of the estimates.

#### Underweight

**Fig 4:**
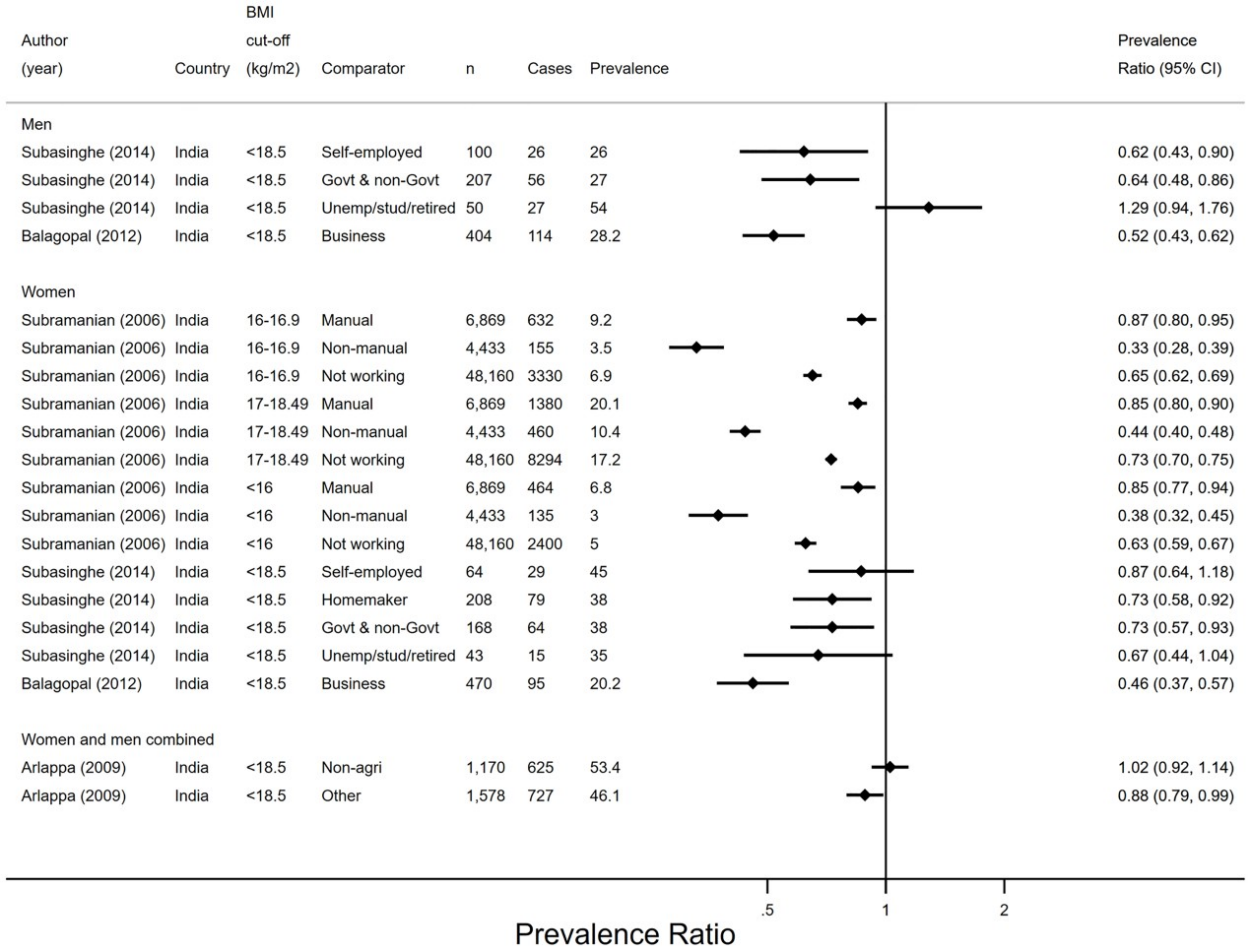
Prevalence ratios and 95% confidence intervals of underweight by employment status (n = 4). Agri–agriculture, BMI–body mass index, CI–confidence interval, Govt–government, n–sample size, PR–prevalence ratio, stud–student. Prevalence ratios were derived from comparing each non-agricultural group (coded 1) to the agricultural group (coded 0).

Four studies presented sufficient data to calculate PRs (95% CIs) for underweight (BMI < 18.5kg/m^2^) (Fig 4). Three studies were set in rural areas and one included rural and urban workers. All studies suggested that farmers had higher prevalence of underweight than homemakers,[36] manual and non-manual women[38]; business,[39] government and non-government[36] women and men; and ‘other’ (than non-agricultural) workers in analyses combining genders.[37] The prevalence of underweight was higher among farming than among self-employed men but not women.[36] Underweight was more prevalent among farmers than among unemployed women,[38] but similar to students, retired and unemployed people (the latter two were combined in gender-specific analyses).[36] There were no differences in the prevalence of underweight between farmers and other (non-specific) non-agricultural workers.[37]

#### Tobacco

**Fig 5:**
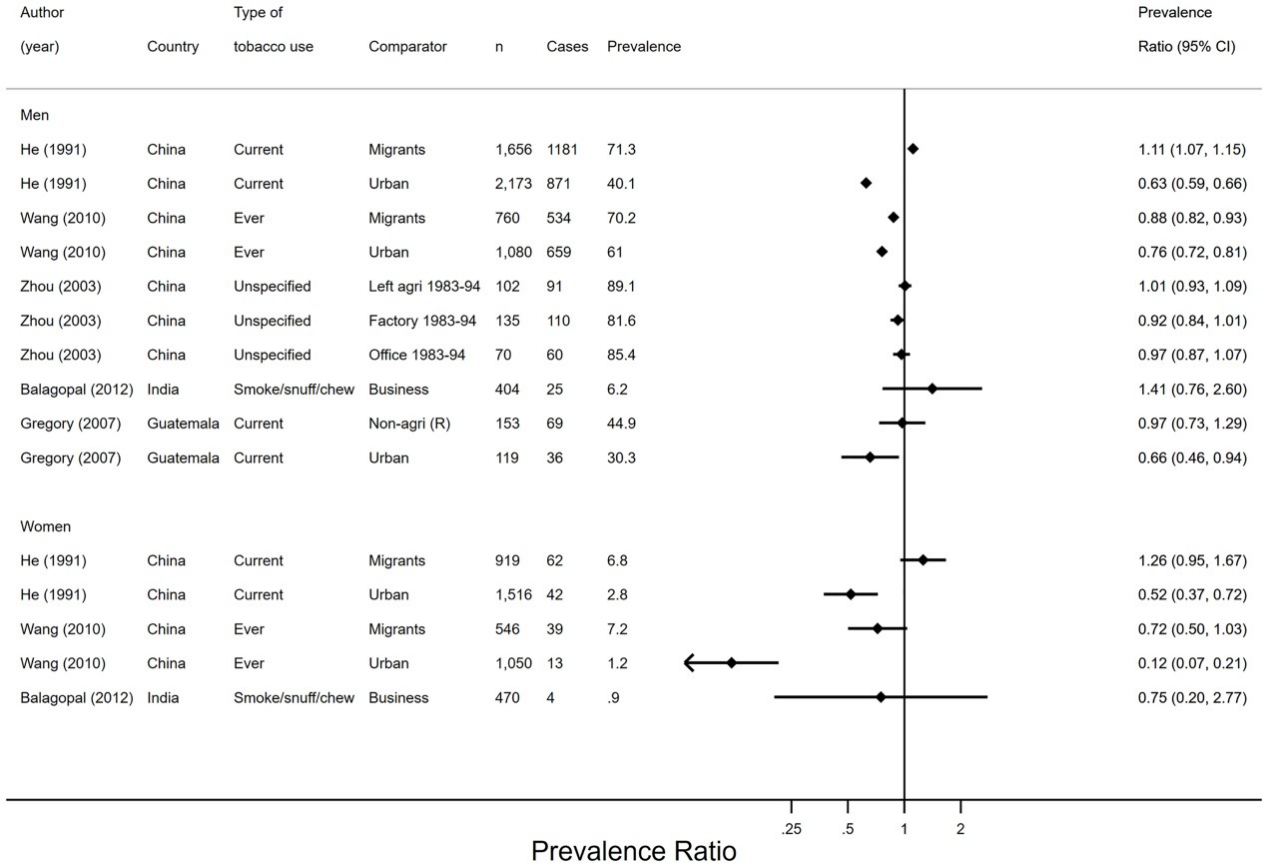
Prevalence ratios and 95% confidence intervals of tobacco use by employment status (n = 5). Agri–agriculture, CI–confidence interval, n–sample size, PR–prevalence ratio, R–rural. Prevalence ratios were derived from comparing each non-agricultural group (coded 1) to the agricultural group (coded 0).

Five studies presented sufficient data to calculate PRs (95% CIs) for current smoking (n = 2), ever smoked (n = 1), unspecified period of smoking (n = 1) and chewing, snuffing and smoking tobacco (n = 1) (Fig 5). Two studies were set in rural areas and three were set in rural and urban areas. In one longitudinal study,[29] the prevalence of smoking decreased over 10 years among men who transitioned out of agriculture and into to unspecified occupations. The prevalence of smoking did not change among men who remained in agriculture, factory or office work over the 10 years.[29] Smoking prevalence did not differ between the four groups at the 10-year follow-up (Fig 5). Farmers were more likely to smoke than urban workers in three publications (from two studies).[32, 40, 41] Two publications used data from different time-points of the same study and found that migrant men were more likely to smoke than farming men in 1991[40] and less likely to smoke than farming men in 2010[41]. Although PRs for migrant versus farming women were similar to those of men in the two studies, they had wide 95% CIs that included the null-value of one. Two studies did not support differences between agricultural workers and non-agricultural rural workers[32] or business workers.[39]

## Discussion

We aimed systematically to review the published evidence on the association of engaging in agriculture compared to other types of non-agricultural employment with atherosclerotic CVDs and over 50 associated modifiable risk factors. Following the protocol, we found two studies on the primary outcomes, atherosclerotic CVDs and 13 studies on four secondary outcomes, hypertension, overweight and obesity, underweight and tobacco use. Included studies were predominantly from India (n = 7) and China (n = 3). Heterogeneity in study settings, populations under study, measurement methods and categorisation of exposures and outcomes prevented formal meta-analyses with pooled results and generation of funnel plots.

Older agricultural workers had a lower CVD mortality rate than retired individuals in a cohort of rural residents from Vietnam, whereas the prevalence of CHD did not differ with employment in a study from rural India. Five out of nine studies suggested that people who engaged in agriculture had a lower prevalence of hypertension than migrant, urban, factory and retired sedentary workers. The evidence suggested no difference or contradictory results on the prevalence of hypertension for a number of other occupations with no clear pattern. One study reported that migrants had a higher likelihood of hypertension than farmers after adjusting for age and gender. Most evidence suggested that people who engaged in agriculture were less likely to be overweight and obese and more likely to be underweight than most non-agricultural workers they were compared to. Urban men and women appeared less likely to smoke than farmers in two studies, whereas results were contradictory for migrants. The prevalence of smoking declined among men transitioning out of agriculture and remained unchanged among men who did not change their occupation during 10 years. However, there were no differences between groups at the 10-year follow-up. The associations of type of labour could not be separated from those of location of residence for any of the outcomes because of the way different types of employment were sampled, for example, rural agricultural workers and urban government workers.

To the best of our knowledge, this is the first systematic review to assess associations of engaging in agriculture compared to types of non-agricultural employment with CVD incidence, prevalence and risk factors. A main contribution of this review was the calculation and comparison of PRs and 95% CIs across studies with available data, including studies that did not perform statistical tests. We attempted to reduce overestimation of associations from studies with high outcome prevalence by calculating PRs (95% CIs), as ORs are prone to overestimate the strength of associations in this context.[45] There are also a number of limitations to this review. A main limitation was the lack of eligible studies, particularly on the primary outcomes, atherosclerotic CVDs. The lack of studies on primary outcomes was likely due to a lack of this type of data from LMIC, e.g. from national surveys and disease surveillance.[3] It is also possible that we missed some relevant studies. The lack of studies on secondary outcomes were more surprising and is likely rooted in a disconnect between sectors and sciences concerned with employment/labour and chronic diseases that are inherently interlinked.[11, 13, 46] A number of studies and risk factors were excluded from the review as a result of restricting analyses to risk factors that were reported in four or more studies and for which clear descriptions of measurement methods and outcome categorisations were provided. Our list of risk factors was comprehensive, but not exhaustive and we may in turn have neglected some important relationships of agriculture and CVD risk factors, e.g. mental health.

Substantial limitations of the included studies made it difficult to draw conclusions. The absence of definitions and descriptions of the measurement methods and categorisations of exposure and comparators complicated the interpretation of results. It may be challenging to measure livelihoods and types of employment in LMIC settings where the informal sector dominates and livelihood diversification and seasonal migration are common.[47] It is possible that the ‘current status’ in cross-sectional studies reflect the work done on the day or season of the survey, at least for some employment groups, e.g. daily wage earners. In turn, some misclassification of results is expected to dilute our results (towards the null). In all studies, participants may have belonged to their current exposure or comparator group for varying lengths of time. Survivor bias arising from ill-health or death from the reviewed risk factors or related CVD events in the time of employment prior to study start may explain some of this review’s inconsistent and contradictory results.[48] To varying degree, healthy worker bias may have diluted and possibly reversed associations, particularly in studies of older adults or other high-risk groups.[48, 49] However, studies of younger adults in LMICs may also be at risk of this kind of bias, as premature deaths and disability from chronic diseases become more common.[3] Some employment groups, e.g. highly physical work, may attract healthier individuals. For example, it is common for young adults in LMICs to migrate from rural to urban areas for work in e.g. construction (men) or housekeeping (women),[47] leaving older, potentially less healthy, adults behind in agriculture.[50] None of the included studies adequately addressed differences between included and excluded participants. Large differences in time between included studies (1991 to 2015) further pose challenges to directly comparing results within and between studies, as well as inferring relevance of the observed associations in the present contexts of LMICs.

We could not determine the extent to which potential confounders might have accounted for observed associations (or absence of associations) because of the lack of appropriately adjusted analyses. An important example is socio-economic status, which particularly may mask results of studies that included or compared rural and urban workers. For example, wealthier urban residents may enjoy health benefits from availability of and access to goods and services, such as food, water, sanitation and health-services. In contrast, the health of the urban poor may be worse than that of the rural poor,[18] for example as a result of unsafe living conditions and high living costs.[47] This may explain some of the inconsistent findings, e.g. among manual workers, business/trade and the unemployed, in studies sourcing participants from different settings. We appreciate that hypothesis-generating studies, that analysed multiple exposure-outcome relationships may have limited their number of analyses to reduce the risk of producing ‘statistically significant’ results by chance. Few studies reported that they based sample sizes on power calculation and wide confidence intervals in several studies suggested that the statistics power might not be high enough to detect existing differences. Missing data may have additionally favoured or diluted associations in several studies. Finally, there were some indications of selective reporting of results from most studies. To some extent, this could be explained by the large scope of several exploratory studies, which may not allow for reporting all analyses. It is also possible that some studies gave preference to ‘statistically significant’ results in order to improve chances of publication.[26]

It is common for LMIC governments, e.g. in Indian and China, to facilitate labour-force shifts out of agriculture and into industry and service sectors to promote economic growth during development.[51, 52] Concurrently, national age-standardised prevalence of death and disability from CVDs have risen and the Employment Conditions Knowledge Network expresses concern that LMICs will be unable to provide the growing urban labour-force with fair employment opportunities.[11, 53] Progress reports on China’s recent urbanisation have shown unexpected challenges with integrating agricultural workers into new types of employment and urban settings. Chinese migrant workers, for example, suffered social exclusion and were denied equal rights to fair employment, housing and health services, which is associated with infectious and chronic diseases.[51, 54] As previously discussed, our search only returned one study that examined the association of employment transitions out of agriculture with cardiovascular health in LMICs. The unexpected developments in China warrant a deeper understanding of how employment transitions are associated with CVDs and risk factors to ensure current economic growth strategies do not add to already expected rises in CVD burdens with development in LMICs.

Evidence on associations of types of employment, and particularly employment transitions, with chronic diseases and related risk factors is fragmented. Where the health sciences typically assess an association of an intervention with an outcome within a single type of employment (e.g. agriculture [55, 56]) the employment sector tends to focus on shorter-term issues relating to health and safety regulation-outcomes (e.g. ergonomics, injuries or exposure to noise or hazardous agents).[11] The data collected across sectors vary widely, for example in relation to sources and collection methods, and are not easily combined for epidemiological studies that can assess health risks or benefits that are associated with types of employment. Prospective longitudinal studies should take advantage of the current momentum for interdisciplinary initiatives across the 2030 agenda for sustainable development, such as ‘decent work and economic development’, ‘sustainable cities and communities’ and ‘health and well-being for all’ in LMICs.[57] New study models and data collection approaches are likely to be required to address these challenging needs. Studies should also address the methodological shortcomings identified in this review by complying with appropriate reporting guidelines,[58] namely ensuring appropriate selection of participants that accommodates conditional comparability of outcomes across exposure and comparator groups; use of appropriate measurement methods for collection of all data; and appropriate reporting of study protocol, methods and results (including the reporting of missing data and ‘non-significant’ findings).

## Conclusion and implications

There was some, however limited, evidence of negative associations of engaging in (rural) agriculture compared to types of (usually urban) employment with prevalence of hypertension, overweight and obesity, and positive associations with prevalence of underweight and smoking. There were no clear patterns indicating which types of employment were associated with higher or lower prevalence of outcomes. High quality evidence is lacking on how engaging in agriculture compared to types of non-agricultural employment may act on CVDs and risk factors in LMICs. Rigorous studies that address the methodological shortcomings identified by this review are needed. They should cultivate a consensus of how best to measure and categorise employment when investigating chronic disease outcomes as well as appropriately control for potential confounders. We further call for new models of interdisciplinary longitudinal studies, for example across health sciences, livelihoods, demography and policy, that assess the association of livelihood transitions and migration with core health outcomes in LMICs. These sorts of studies should seek to inform evidence-based chronic disease prevention measures in national economic growth strategies to safeguard health during development in line with the sustainable development goals.

## Supporting information

S1 Checklist PRISMA checklist. (DOC)

S1 Table Search strategy. (DOCX)

S2 Table Characteristics of studies excluded from results synthesis (n = 11). BMI–Body Mass Index; CHD–Coronary Heart Disease; cm–centimetre(s); CVD Cardiovascular Disease; d–day; DBP–diastolic blood pressure; EE–energy expenditure; g–gram; HDL–High-Density Lipoprotein cholesterol; HH–household; kcal–kilocalories; kg–kilograms; KJ—kilojoule; LDL–Low-Density Lipoprotein cholesterol; LMIC–low- and middle-income country; m–metre(s); mg–milligram; min–minute; mmHg–millimetre mercury; n–sample size; N/A–not available; PA–physical activity; SBP–systolic blood pressure; TC–total cholesterol; TG–triglycerides; WC–waist circumference; WHO–World Health Organization; WHR–waist-to-hip ratio; % BF–percent body fat; μg–microgram.
(DOCX)

S3 Table Detailed characteristics of included studies (n = 13). BMI–Body Mass Index; CED–Chronic Energy Deficiency; HH–household(s); INCAP–Nutrition of Central America and Panama longitudinal study; kg–kilograms; l–litre; m–metre(s); mmHg–millimetre mercury; mmol–millimoles; n–sample size; N/A–not available; PA–physical activity; SBP–systolic blood pressure; YMS–Yi Migrant Study.
(DOCX)

S4 Table Quality of evidence rating of included studies from five countries (n = 13). ^a^The risk of bias assessment was based on adapted ‘A Cochrane Risk of Bias Assessment Tool: for Non-Randomized Studies of Interventions.
(DOCX)

## Abbreviations

% BF: percent body fat
Agri: agriculture
BMI: body mass index
BMI: body mass index
CHD: coronary heart disease
CI: confidence interval
Cm: centimetre(s)
CVD: cardiovascular disease
CVD: cardiovascular disease
d: day
DBP: diastolic blood pressure
EE: energy expenditure
g: gram
Govt: government
HDL: high-density lipoprotein cholesterol
HH: household
HH: household(s)
INCAP: Nutrition of Central America and Panama longitudinal study
Kcal: kilocalories
Kg: kilograms
KJ: kilojoule
l: litre
LDL: low-density lipoprotein cholesterol
LMIC: Low- and middle-income country
LMIC: low- and middle-income country
LSHTM: London School of Hygiene and Tropical Medicine
m: metre(s)
mg: milligram
min: minute
mmHg: millimetre mercury
mmol: millimoles
N: sample size
n: sample size
N/A: not available
NCD: non-communicable disease
OR: odds ratio
PA: physical activity
PICOS: participants, interventions, comparisons, outcomes, and study design
PR: prevalence ratio
PRISMA: Preferred Reporting Items for Systematic Reviews and Meta-Analyses
R: rural
SBP: systolic blood pressure
Stud: student
TC: total cholesterol
TG: triglycerides
UK: United Kingdom
WC: waist circumference
WHO: World Health Organization
WHR: waist-to-hip ratio
YMS: Yi Migrant Study
μg: microgram
