# Supplementary material for: Is agricultural engagement associated with lower incidence or prevalence of cardiovascular diseases and cardiovascular disease risk factors? A systematic review of observational studies from low- and middle-income countries
Source: PLoS One. 2020 Mar 31;15(3):e0230744. doi: 10.1371/journal.pone.0230744 (PMC7108743; doi:10.1371/journal.pone.0230744)
Supplement: S1 Table — (DOCX) [file pone.0230744.s002.docx]

S2 Table Search strategy

| **Search number (#) and description** |
| --- |
| #1 Search (animal husbandry/ OR aquaculture/ OR beekeeping/ OR dairying/ OR gardening/ OR organic agriculture/[MeSH Terms])  #2 Search (agri*[Title/Abstract] OR agrarian[Title/Abstract] OR agro*[Title/Abstract] OR farm*[Title/Abstract] OR fishing[Title/Abstract] OR aquaculture[Title/Abstract] OR fisherm#n[Title/Abstract] OR mariculture[Title/Abstract] OR pisciculture[Title/Abstract] OR pastoral[Title/Abstract] OR livestock[Title/Abstract] OR dairying[Title/Abstract] OR floriculture*[Title/Abstract] OR horticulture*[Title/Abstract] OR forestry[Title/Abstract])  #3 Search (“Animal husbandry”[Title/Abstract] OR “animal rearing”[Title/Abstract] OR “livestock rearing”[Title/Abstract] OR “crop production”[Title/Abstract] OR “food production”[Title/Abstract] OR “animal production”[Title/Abstract] OR “poultry production”[Title/Abstract] OR “swine production”[Title/Abstract] OR “livestock production[Title/Abstract] OR “poultry farm*”[Title/Abstract] OR “swine farm*”[Title/Abstract])  #4 Search (land-use[Title/Abstract] OR land-own*[Title/Abstract] OR pasture[Title/Abstract])  #5 Search (“Arable land”[Title/Abstract] OR “agricultur* land”[Title/Abstract] OR “farm land”[Title/Abstract] OR “own* land”[Title/Abstract] OR ”land use*”[Title/Abstract])  #6 Search ((((#1) OR #2) OR #3) OR #4) OR #  #7 Search (cardiovascular diseases/ OR myocardial ischemia/ OR pulmonary heart disease/ OR brain ischemia/ OR carotid artery thrombosis/ OR stroke, lacunar/ OR cerebral arterial diseases/ OR intracranial arteriosclerosis/ OR intracranial embolism and thrombosis/ OR intracranial thrombosis/ OR stroke/ OR embolism and thrombosis/ OR hypertension/ OR hypotension/ OR peripheral vascular diseases/ OR prehypertension/ OR poisoning/ OR substance-related disorders/[MeSH Terms])  #8 Search (Stroke [Title/Abstract] OR “coronary heart disease” [Title/Abstract] OR CHD[Title/Abstract] OR “cardio-vascular”[Title/Abstract] OR CVD[Title/Abstract] OR “heart disease?”[Title/Abstract] OR “cardiometabolic”[Title/Abstract]  #9 Search (exp body fat distribution/[Title/Abstract] OR body mass index/[Title/Abstract] OR waist circumference/[Title/Abstract] OR waist-height ratio/[Title/Abstract] OR cholesterol/[Title/Abstract] OR apolipoproteins/[Title/Abstract] OR exp apolipoproteins a/[Title/Abstract] OR exp apolipoproteins b/OR Triglycerides/[Title/Abstract] OR exp Hypertriglyceridemia/[Title/Abstract] OR diabetes mellitus, type 2/[Title/Abstract] OR diabetes, gestational/[Title/Abstract] OR diabetic ketoacidosis/[Title/Abstract] OR prediabet* state/[Title/Abstract] OR hyperglycemia/[Title/Abstract] OR glucose intolerance/[Title/Abstract] OR insulin resistance/[Title/Abstract] OR metabolic syndrome x/[Title/Abstract] OR Hyperinsulinism/[Title/Abstract] OR Vascular Stiffness/[Title/Abstract] OR C-Reactive Protein/[Title/Abstract] OR Carotid Intima-Media Thickness/[Title/Abstract] OR Homocysteine/[Title/Abstract] OR exp *Dyslipidemias/[Title/Abstract] OR *Alcohols/[Title/Abstract] OR dietary fats/[Title/Abstract] OR cholesterol, dietary/[Title/Abstract] OR *fatty acids/[Title/Abstract] OR Trans Fatty Acids/[Title/Abstract] OR Dietary Carbohydrates/[Title/Abstract] OR Antioxidants/[Title/Abstract] OR Vitamin E/[Title/Abstract] OR Ascorbic Acid/[Title/Abstract] OR Ubiquinone/[Title/Abstract] OR exp Flavonoids/[Title/Abstract] OR exp carotenoids/[Title/Abstract] OR beta carotene/[Title/Abstract] OR Selenium/[Title/Abstract] OR Folic Acid/[Title/Abstract] OR Vitamin B Complex/[Title/Abstract] OR exp Fatty Acids, Omega-3/[Title/Abstract] OR sodium chloride, dietary/OR Dietary Fibre/[Title/Abstract] OR exp Fruit/[Title/Abstract] OR Vegetables/[Title/Abstract] OR Exercise/[Title/Abstract])  #10 Search (“Body mass index”[Title/Abstract] OR BMI[Title/Abstract] OR “waist circumference”[Title/Abstract] OR “hip circumference”[Title/Abstract] OR “waist-to-hip ratio”[Title/Abstract] OR WHR[Title/Abstract] OR “body fat”[Title/Abstract] OR “fat percentage”[Title/Abstract] OR “blood pressure”[Title/Abstract] OR hypertension[Title/Abstract] OR cholesterol[Title/Abstract] OR HDL[Title/Abstract] OR LDL[Title/Abstract] OR Apolipoprotein*[Title/Abstract] OR triglycerides[Title/Abstract] OR hypertriglycerid?emia[Title/Abstract] OR diabetes[Title/Abstract] OR DMTII[Title/Abstract] OR DMT2[Title/Abstract] OR TIIDM[Title/Abstract] OR T2DM[Title/Abstract] OR hyperglyc?emia[Title/Abstract] OR “impaired fasting glucose”[Title/Abstract] OR “insulin resistance”[Title/Abstract] OR hyperinsulin*[Title/Abstract] OR “Homeostatic Model Assessment”[Title/Abstract] OR HOMA[Title/Abstract] OR “augmentation index”[Title/Abstract] OR AIX[Title/Abstract] OR “arterial stiffens”[Title/Abstract] OR “C-reactive protein”[Title/Abstract] OR CRP[Title/Abstract] OR “hs-CRP”[Title/Abstract] OR “carotid intima-media thickness”[Title/Abstract] OR CIMT[Title/Abstract] OR IMT[Title/Abstract] OR homocysteine[Title/Abstract] OR Framingham[Title/Abstract] OR dyslipid?emia[Title/Abstract] OR “metabolic syndrome”[Title/Abstract] OR “syndrome x”[Title/Abstract])  #11 Search (alcohol*[Title/Abstract] OR beer[Title/Abstract] OR spirit[Title/Abstract] OR wine[Title/Abstract] OR fat[Title/Abstract] OR “trans-fat”[Title/Abstract] OR carbohydrate[Title/Abstract] OR sugar[Title/Abstract] OR antioxidant*[Title/Abstract] OR vitamin E[Title/Abstract] OR Vitamin C[Title/Abstract] OR Ascorbic acid[Title/Abstract] OR ubiquinone[Title/Abstract] OR coenzyme Q[Title/Abstract] OR bioflavonoids[Title/Abstract] OR “beta-carotene”[Title/Abstract] OR selenium[Title/Abstract] OR “folic acid”[Title/Abstract] OR “vitamin B6”[Title/Abstract] OR “vitamin B12”[Title/Abstract] OR “omega-3”[Title/Abstract] OR “n-3 fat*”[Title/Abstract] OR salt[Title/Abstract] OR sodium[Title/Abstract] OR fibre[Title/Abstract] OR fruit?[Title/Abstract] OR vegetable?[Title/Abstract] OR diet[Title/Abstract] OR “physical activity”[Title/Abstract] OR PAL[Title/Abstract] OR “physically active”[Title/Abstract] OR sedentary[Title/Abstract] OR inactiv*[Title/Abstract] OR “energy expenditure”[Title/Abstract] OR “metabolic equivalent?”[Title/Abstract] OR MET?[Title/Abstract] OR “physical activity ratio”[Title/Abstract] OR PAR?[Title/Abstract])  #12 Search (β-carotene[Title/Abstract] OR ω-3 fat*[Title/Abstract] OR omega?3 fat*[Title/Abstract])  #13 Search (((((#7) OR #8) OR #9) OR #10) OR #11) OR #12  #14 Search Developing Countries/[MeSH Terms]  #15 Search (“Low-and middle income countr*”[Title/Abstract] OR lmic*[Title/Abstract] OR “third world”[Title/Abstract] OR “lami countr*”[Title/Abstract] OR “transitional countr*”[Title/Abstract] OR “developing econom*”[Title/Abstract] OR “less* developed econom*”[Title/Abstract] OR “under-developed econom*”[Title/Abstract] OR “underdeveloped econom*”[Title/Abstract] OR “middle income econom*”[Title/Abstract] OR “low* income econom*”[Title/Abstract] OR “developing countr*”[Title/Abstract] OR “less* developed countr*”[Title/Abstract] OR “under-developed countr*”[Title/Abstract] OR “underdeveloped countr*”[Title/Abstract] OR “middle income countr*”[Title/Abstract] OR “low* income countr*”[Title/Abstract] OR “underserved countr*”[Title/Abstract] OR “under served countr*”[Title/Abstract] OR “deprived countr*”[Title/Abstract] OR “poor* countr*”[Title/Abstract] OR “developing nation?”[Title/Abstract] OR “less* developed nation?”[Title/Abstract] OR “under-developed nation?”[Title/Abstract] OR “underdeveloped nation?”[Title/Abstract] OR “middle income nation?”[Title/Abstract] OR “low* income nation?”[Title/Abstract] OR “underserved nation?”[Title/Abstract] OR “under served nation?”[Title/Abstract] OR “deprived nation?”[Title/Abstract] OR “poor* nation?”[Title/Abstract] OR “developing population?”[Title/Abstract] OR “less* developed population?”[Title/Abstract] OR “under-developed population?”[Title/Abstract] OR “underdeveloped population?”[Title/Abstract] OR “middle income population?”[Title/Abstract] OR “low* income population?”[Title/Abstract] OR “underserved population?”[Title/Abstract] OR “under served population?”[Title/Abstract] OR “deprived population” [Title/Abstract] OR “poor* population?”[Title/Abstract] OR “developing world”[Title/Abstract] OR “low* gdp”[Title/Abstract] OR “low* gnp”[Title/Abstract] OR “low* gross domestic”[Title/Abstract] OR “low* gross national”[Title/Abstract] OR Asia[Title/Abstract] OR India[Title/Abstract] OR “Andhra Pradesh”[Title/Abstract])  #16 Search (#14) OR #15  #17 Search ((#6) AND #13) AND #16  #18 Search ((#6) AND #13) AND #16 Filters: Humans |
