# Supplementary material for: Is agricultural engagement associated with lower incidence or prevalence of cardiovascular diseases and cardiovascular disease risk factors? A systematic review of observational studies from low- and middle-income countries
Source: PLoS One. 2020 Mar 31;15(3):e0230744. doi: 10.1371/journal.pone.0230744 (PMC7108743; doi:10.1371/journal.pone.0230744)
Supplement: S2 Table — BMI–Body Mass Index; CHD–Coronary Heart Disease; cm–centimetre(s); CVD Cardiovascular Disease; d–day; DBP–diastolic blood pressure; EE–energy expenditure; g–gram; HDL–High-Density Lipoprotein cholesterol; HH–household; kcal–kilocalories; kg–kilograms; KJ—kilojoule; LDL–Low-Density Lipoprotein cholesterol; LMIC–low- and middle-income country; m–metre(s); mg–milligram; min–minute; mmHg–millimetre mercury; n–sample size; N/A–not available; PA–physical activity; SBP–systolic blood pressure; TC–total cholesterol; TG–triglycerides; WC–waist circumference; WHO–World Health Organization; WHR–waist-to-hip ratio; % BF–percent body fat; μg–microgram. (DOCX) [file pone.0230744.s003.docx]

S3 Table Characteristics of studies excluded from results synthesis (n=11)

| **Author and Year** | **Country** | **Population and site** | **Study design, sampling  (n)** | **Relevant outcomes** | **Relevant outcome(s) measured but not reported by employment** | **Covariates** | **Exposure and comparator(s)** | **Participants** | | | | | | **Age range, years** | **Comments** |
| --- | --- | --- | --- | --- | --- | --- | --- | --- | --- | --- | --- | --- | --- | --- | --- |
|  |  |  |  |  |  |  |  | **n (%)** | | | **Age, mean (SD)** | | |  |  |
|  |  |  |  |  |  |  |  | **Men** | **Women** | **Total** | **Men** | **Women** | **Total** |  |  |
| Atkinson et al. 2016 | 47 low- and middle-income countries (LMIC) | Urban and rural areas | Cross-sectional (on data from World Health Organization (WHO) Health Surveillance Information Study (n=70 countries)), selected on health surveillance needs  (n=242,753) | **Excluded:** Physically inactive (International Physical Activity Questionnaire Score) | None | **Country level:** Human Development Index, economic development, urbanisation   **Individual level:** age, gender, education, income, rural/urban residence | Agriculture |  |  |  |  |  |  | 18, 69 |  |
|  |  |  |  |  |  |  | White-collar |  |  |  |  |  |  |  |  |
|  |  |  |  |  |  |  | Blue-collar |  |  |  |  |  |  |  |  |
|  |  |  |  |  |  |  | Homemaker |  |  |  |  |  |  |  |  |
|  |  |  |  |  |  |  | Unemployed |  |  |  |  |  |  |  |  |
|  |  |  |  |  |  |  | Total |  |  | 196,742 |  |  |  |  |  |
| Barker et al. 2006 | India | Rural village in agricultural community near Pune (Paba) | Cross-sectional, families from the Maternal Nutrition and Fetal Growth Study (n=797), non-random sample of married women in the village  (n=180) | **Excluded:** Body Mass Index (BMI) (kilograms (kg)/metres (m)^2^) | Fruit consumption | None | Farming | 63 | 79 |  |  |  |  | Reproductive age | Husband and wife pairs of child bearing age with at least one son and one daughter aged three to eight years (mainly agricultural castes) |
|  |  |  |  |  |  |  | Non-farming | 30 | 19 |  |  |  |  |  |  |
| Gupta et al. 1997 | Rajasthan, India | Three villages in rural communities (Bagoth, Badoo and Janjila in Parbatsar Tehsil, county of Nagaur) | Cross-sectional,  entire communities in randomly selected villages  (n=3,148) | **Excluded:** Coronary Heart Disease (CHD) | Alcohol, diastolic blood pressure (DBP), family history of diabetes and CHD and High-Density Lipoprotein (HDL), history of diabetes, tobacco, physical activity (PA); hypertension, Low-Density Lipoprotein (LDL), obesity, systolic blood pressure (SBP), total cholesterol (TC), triglycerides (TG), waist-to-hip-ratio (WHR) | Age, gender, education, anger/grief, prayer habit, family structure, religion, marital status, vegetarianism and intake of Ghee, (depression, stressful life events, housing and Yoga practice) | Agriculture | 1,303 | 180 | 1,483 |  |  |  | >20 | Desert population. Engage in farming activities for two to three months per year   Low response rate for women (59.2%) due to Purdah custom (religious seclusion of women) |
|  |  |  |  |  |  |  | Business | 377 | 19 | 396 |  |  |  |  |  |
|  |  |  |  |  |  |  | Professional | 4 | 3 | 7 |  |  |  |  |  |
|  |  |  |  |  |  |  | Government | 298 | 31 | 329 |  |  |  |  |  |
|  |  |  |  |  |  |  | Household | 0 | 933 | 933 |  |  |  |  |  |
|  |  |  |  |  |  |  | Total | 1,982 | 1,166 | 3,148 | 39.87 (15) | 36.81 (3) | 38.73 (14) |  |  |
| Panwar & Punia 1998a | Orissa,  India | Pregnant women from six rural farming/non-farming villages in Haryana State | Cross-sectional, sampling methods not available (N/A)  (n=90) | **Excluded:** fruit consumption (grams (g)/day (d)), vegetable consumption (g/d) | None | None | Farming | 0 | 45 |  |  |  |  | Reproductive age | Pregnant women |
|  |  |  |  |  |  |  | Non-farming | 0 | 45 |  |  |  |  |  |  |
| Panwar & Punia 1998b | Orissa,  India | Pregnant women from six rural farming/non-farming villages in Haryana State | Cross-sectional, sampling methods N/A   (n=90) | Excluded: ascorbic acid (milligrams (mg)/d), folic acid (micrograms (µg)/d), riboflavin (mg/d) | None | None | Farming | 0 | 45 |  |  |  |  | Reproductive age | Pregnant women |
|  |  |  |  |  |  |  | Non-farming | 0 | 45 |  |  |  |  |  |  |
|  |  |  |  |  |  |  |  |  |  |  |  |  |  |  |  |
| Poulter et al. 1984 | Nyanza Province, Kenya | 35 rural villages in Siaya District | Cross-sectional,  random sample of Lou tribe (rural) and volunteers of potential (Lou) migrants (rural)  (n=2,334) | **Excluded:** BMI (kg/m^2^), DBP (millimetre mercury (mmHg)) (age-adjusted), SBP (mmHg) (age-adjusted) | None | Age, education, sodium level, potassium level, sodium:potasium ratio, weight | Land work |  |  |  |  |  |  | ≥17 | Occupation data missing for 37% of participants  Combines participants sampled by two methods (861 men and women from census and 1473 male volunteers) |
|  |  |  |  |  |  |  | Other work (teaching, machinery maintenance, lorry driving etc.) |  |  |  |  |  |  |  |  |
|  |  |  |  |  |  |  | Total |  |  | 2,334 |  |  |  |  |  |
| Pritchard et al. 2016 | Himachal Pradesh and Uttarakhand, India | Two (anonymous) rural villages, on each side of the boundary between Himachal Pradesh and Uttarakhand | Cross-sectional (of cohort), three-stage households (HH) baseline random survey strategy (purpose sampling of villages)  (n=123 HHs) | **Excluded:** fruits (kilojoules (KJ)/d), vegetables (KJ/d) | None | None | HH farming own plot |  |  |  |  |  |  | Adult HH members, adjusted for children | Men, women and children (results are adjusted for infants and children)  Villages differ markedly in history of landholding, social patterning of land and food insecurity |
|  |  |  |  |  |  |  | HH farming other’s land/ share-cropping |  |  |  |  |  |  |  |  |
|  |  |  |  |  |  |  | HH with one or more non-agricultural livelihood sources |  |  |  |  |  |  |  |  |
|  |  |  |  |  |  |  | HH farming own plot and one or more non-farm livelihood sources |  |  |  |  |  |  |  |  |
|  |  |  |  |  |  |  | Total | 123 HHs |  |  |  |  |  |  |  |
| Sengupta 2014 | Andhra Pradesh and West Bengal, India | Fishermen from rural Araku valley of Visakhapatnam District, Andhra Pradesh and urban college students from Kolkata, West Bengal | Cross-sectional, random sample (site selection N/A)  (n=50) | **Excluded:** BMI (kg/m^2^), DBP (mmHg), energy expenditure (EE) (kcal minute (min)^-2^), % Body fat (% BF) (%) (age adjusted), SBP (mmHg), waist circumference (WC) (centimetres (cm)), WHR | None | Age | Fishermen | 25 | 0 |  | 22.8 (1.92) |  |  |  | Non-smokers  Students are different samples from the same college as other studies by Sengupta and colleagues |
|  |  |  |  |  |  |  | College students | 25 | 0 |  | 21.9 (2.25) |  |  |  |  |
|  |  |  |  |  |  |  |  |  |  |  |  |  |  |  |  |
|  |  |  |  |  |  |  |  |  |  |  |  |  |  |  |  |
|  |  |  |  |  |  |  |  |  |  |  |  |  |  |  |  |
|  |  |  |  |  |  |  |  |  |  |  |  |  |  |  |  |
|  |  |  |  |  |  |  |  |  |  |  |  |  |  |  |  |
| Sengupta & Sahoo 2013 | Orissa and West Bengal, India | Fishermen slum in rural Puri, Orissa and urban college students from Kolkata, West Bengal | Cross-sectional, random sample (site selection N/A)  (n=30) | **Excluded:** BMI (Kg/m^2^), DBP (mmHg), EE (kcal min^-2^), % BF (%) (age adjusted), SBP (mmHg) | None | Age | Fishermen (slum) | 15 | 0 |  | 22.2 (2.70) |  |  | 18, 25 | Non-smokers  Students are different samples from the same college as other studies by Sengupta and colleagues |
|  |  |  |  |  |  |  | College students | 15 | 0 |  | 21.0 (2.25) |  |  |  |  |
|  |  |  |  |  |  |  |  |  |  |  |  |  |  |  |  |
|  |  |  |  |  |  |  |  |  |  |  |  |  |  |  |  |
|  |  |  |  |  |  |  |  |  |  |  |  |  |  |  |  |
| Sengupta & Sahoo 2011 | West Bengal, India | Fishermen slum in rural East Midnapore and urban college students from Kolkata, West Bengal | Cross-sectional random sampling (site selection N/A)  (n=30) | **Excluded:** BMI (kg/m^2^), DBP (mmHg), EE (Kcal min-2), % BF (%) (age adjusted), SBP (mmHg), WC (cm), WHR | None | Age | Fishermen (slum) | 15 | 0 |  | 22.5 (2.97) |  |  | 18, 25 | Non-smokers  Students are different samples from the same college as other studies by Sengupta and colleagues |
|  |  |  |  |  |  |  | College students | 15 | 0 |  | 21.9 (2.16) |  |  |  |  |
| Van Minh et al. 2003 | Hatay Province, Vietnam | Rural areas in Bavi District | Cohort (longitudinal demographic surveillance system FilaBavi (n=49,543 person-years)), Village cluster design   (n=15,193 person-years) | **Excluded:** Cardiovascular Disease mortality (CVD) (for subgroup) | CHD mortality, CVD mortality, pulmonary heart disease mortality, stroke mortality | Age, gender, education, economic condition | Farmer |  |  |  |  |  |  | 50, >70 | Analysis restricted to subsample of participants aged ≥50 years (n=15 193/49 543 person-years) |
|  |  |  |  |  |  |  | Government |  |  |  |  |  |  |  |  |
|  |  |  |  |  |  |  | Other |  |  |  |  |  |  |  |  |
|  |  |  |  |  |  |  | Non-pension retired |  |  |  |  |  |  |  |  |
|  |  |  |  |  |  |  | Total |  |  | 15,193 person- years |  |  |  |  |  |
