# Supplementary material for: Is agricultural engagement associated with lower incidence or prevalence of cardiovascular diseases and cardiovascular disease risk factors? A systematic review of observational studies from low- and middle-income countries
Source: PLoS One. 2020 Mar 31;15(3):e0230744. doi: 10.1371/journal.pone.0230744 (PMC7108743; doi:10.1371/journal.pone.0230744)
Supplement: S3 Table — BMI–Body Mass Index; CED–Chronic Energy Deficiency; HH–household(s); INCAP–Nutrition of Central America and Panama longitudinal study; kg–kilograms; l–litre; m–metre(s); mmHg–millimetre mercury; mmol–millimoles; n–sample size; N/A–not available; PA–physical activity; SBP–systolic blood pressure; YMS–Yi Migrant Study. (DOCX) [file pone.0230744.s004.docx]

S4 Table Detailed characteristics of included studies (n=13)

| **Author and Year** | **Population and site** | **Study design and sampling (n)** | **Outcomes** | **Covariates** | **Exposure and comparators** | **Participants** | | | | | | **Age range, years** | **Comments** |
| --- | --- | --- | --- | --- | --- | --- | --- | --- | --- | --- | --- | --- | --- |
|  |  |  |  |  |  | **n (%)** | | | **Age, mean (SD)** | | |  |  |
|  |  |  |  |  |  | **Men** | **Women** | **Total** | **Men** | **Women** | **Total** |  |  |
| Addo et al. 2006 | Ghana, four rural farming communities (Sarpeiman, Opah, Ayikai Doblo and Amamoley) | Cross-sectional, purpose sampling   (n=362) | Hypertension (≥140/90 millimetre mercury (mmHg)) | Age, gender, education, smoking, alcohol, contraceptive use, work-related physical activity (PA), occupation, Body Mass Index (BMI), diabetes | Farmer |  |  | 107 |  |  | 42.4 (18.6) | 18, 99 | 73% of respondents have physically active employment and do other activities several times/ week |
|  |  |  |  |  | Trader |  |  | 152 |  |  |  |  |  |
|  |  |  |  |  | Other |  |  | 103 |  |  |  |  |  |
|  |  |  |  |  | Total | 107 | 255 | 362 |  |  |  |  |  |
| Arlappa et al. 2009 | India, rural areas in nine states | Cross-sectional (of a rapid population-based cross-sectional study by the National Institute of Nutrition and the National Nutrition Monitoring Bureau),  multistage random sampling  (n=1,569) | Chronic energy deficiency (CED) (BMI <18.5 kilograms (kg)/metres (m)^2^) | Caste, age pension, Annapurna, food for work program, acres of land | Agriculture |  |  | 399 |  |  |  | 60, 70+ | Subgroup (n=1,569/3,147 participants aged 18-70 years+); severely drought-affected districts; co-interventions offered to the most vulnerable |
|  |  |  |  |  | Non-agriculture |  |  | 1,170 |  |  |  |  |  |
| Asgary et al. 2013 | Jamkhed,  India, six villages in rural farming community | Cross-sectional,  proportional random sampling by village  (n=224) | Hypertension (≥140/90 mmHg) | Age, gender, occupation, income, abdominal girth, tobacco, alcohol | Farmer |  |  | 112 (52.8) |  |  |  | 40, 85 | The farming area received health interventions |
|  |  |  |  |  | Housekeeper |  |  | 100 (47.1) |  |  |  |  |  |
|  |  |  |  |  | Total | 95 | 129 |  |  |  |  |  |  |
| Balagopal et al. 2012 | Gujarat,  India, rural community | Cross-sectional (baseline of cohort),  exhaustive (all village residents except migrants)  (n=1,638) | Hypertension (systolic blood pressure (SBP) ≥140 mmHg); underweight, overweight, obese (BMI <18.5; 23-24.99; ≥25 kg/m^2^); Tobacco | None | Agrarian (low socio-economic status) | 362 | 402 | 764 | 43.3 (16.1) | 43.3 (15.8) | 43.4 (15.9) | 18+ | Migrant workers (1/5 of population) were not present and thus excluded |
|  |  |  |  |  | Business (high socio-economic status ) | 404 | 470 | 874 | 40.1 (15.5) | 40.4 (15.9) | 40.2 (15.7) |  |  |
| Gregory et al. 2007 | Guatemala, participants of the Institute of Nutrition of Central America and Panama (INCAP) Longitudinal Study (1969–1977) (n=2392), born in four rural villages | Cross-sectional, non-random sample of available INCAP participants  (n=360) | hypertension (≥130/85 mmHg); overweight (BMI ≥25 kg/m^2^), obese (BMI ≥30 kg/m^2^), smoker | Age, gender | Agriculture rural | 88 (24.4) |  |  | 31.7 (4.4) |  |  |  | High rates of infectious disease mortality and migration |
|  |  |  |  |  | Non-agriculture rural | 153 (42.5) |  |  | 31.4 (4.2) |  |  |  |  |
|  |  |  |  |  | Urban | 119 (33.1) |  |  | 33.6 (4.3) |  |  |  |  |
| Hazarika et al. 2004 | Assam, India, native rural population from 25 villages | Cross-sectional, cluster random sampling of households (HHs) (districts selected on geographical location) in one state  (n=3,180) | Hypertension (≥140/90 mmHg) | Age, sex, marital status, extra salt intake, alcohol intake, smoking, BMI, WHR, tobacco chewing, type of work | Service |  |  |  |  |  |  | ≥30 |  |
|  |  |  |  |  | Business |  |  |  |  |  |  |  |  |
|  |  |  |  |  | Cultivator |  |  |  |  |  |  |  |  |
|  |  |  |  |  | Daily wager |  |  |  |  |  |  |  |  |
|  |  |  |  |  | Unemployed |  |  |  |  |  |  |  |  |
|  |  |  |  |  | Others |  |  |  |  |  |  |  |  |
|  |  |  |  |  | Total | 1,441 (45) | 1,739 (55) | 3,180 (100) |  |  |  |  |  |
| He et al. 1991 | Sichuan Province, China, Yi farmers from remote mountain areas, Yi migrants and Han people living ≥ five years in Xichang City and county seats of Butuo, Meigu, and Zhaojue | Cross-sectional (of Yi Migrant Study (YMS)), Cluster randomised with probability proportional to size  (n=14,505) | Hypertension I (140-159/90-94 mmHg) (age standardised), hypertension II (≥160/95) (age standardised); smoking | Age, BMI, smoking, alcohol, heart rate | Farmer | 5,023 | 3,218 |  | 30.9 | 31.8 |  | 15, 89 | Married women usually moved to husband’s village; Yi people are an ethnic minority. Farmers reside in remote mountain areas (altitude ≥1,500 m) with primitive life-styles |
|  |  |  |  |  | Migrant | 1,656 | 919 |  | 34.8 | 31.3 |  |  |  |
|  |  |  |  |  | Urban | 2,173 | 1,516 |  | 34.8 | 32.9 |  |  |  |
| Norboo et al. 2015 | Jammu and Kashmir,  India, two groups from Leh town subdivision, 41 villages representative of six rural subdivisions: Leh block (n=12), Nubra (n=7), Kargil (n=6), Sham (Khalse), Zanskar (n=10), Changthang (n=6) | Cross-sectional,  two-stage stratified sampling (on urban/rural)  (n=2,800) | Hypertension (≥140/90 mmHg); overweight (BMI ≥25 kg/m2) | Age, gender, obesity, rural/urban residence | Farmer |  |  | 1,247 |  |  |  | 20, 94 | Rural participants are volunteers |
|  |  |  |  |  | Nomad |  |  | 220 |  |  |  |  |  |
|  |  |  |  |  | Sedentary worker |  |  | 549 |  |  |  |  |  |
|  |  |  |  |  | **Other, including:** |  |  | 784 |  |  |  |  |  |
|  |  |  |  |  | *Housewife* |  |  | *325* |  |  |  |  |  |
|  |  |  |  |  | *Manual labourer* |  |  | *63* |  |  |  |  |  |
|  |  |  |  |  | *Monk* |  |  | *157* |  |  |  |  |  |
|  |  |  |  |  | *No job* |  |  | *138* |  |  |  |  |  |
|  |  |  |  |  | *Retired sedentary* |  |  | *101* |  |  |  |  |  |
|  |  |  |  |  | Total |  |  | *2,800* |  |  | 53.8 (15.0) |  |  |
| Olugbile & Oyemade 1982 | Two states, Nigeria, workers from two rural areas in two states: an agricultural company and a cement factory | Cross-sectional,  random sampled from Agricultural Production and Supply Company (farmers), Factory workers sampled by ‘stratified method’  (n=276) | Hypertension (≥140/90 mmHg) |  | Agriculture company |  |  | 112 |  |  |  | 20, 59 | Working population; farmer, but not cement workers, may have received health interventions; farmers >59 years were excluded |
|  |  |  |  |  | Factory worker |  |  | 136 |  |  |  |  |  |
| Subasinghe et al. 2014 | Andhra Pradesh, India, 12 rural villages surrounding the Rishi Valley Rural Health Centre, North Western region of Chittoor District | Cross-sectional,  purpose sampling (those who presented at health centre after contact)  (n=1,169) | CED (BMI <18 kg/m^2^) | Age education, HH income, dietary energy | Non-government, government employee | 170 | 206 |  | . |  |  | 18, 55+ | (Partially) disadvantaged population; primarily low income subsistence farmers (excluded landowners not working on land) |
|  |  |  |  |  | Self-employed | 65 | 100 |  |  |  |  |  |  |
|  |  |  |  |  | Farming and livestock | 156 | 170 |  |  |  |  |  |  |
|  |  |  |  |  | Homemaker | 208 | 1 |  |  |  |  |  |  |
|  |  |  |  |  | Unemployed, student, retired | 43 | 50 |  |  |  |  |  |  |
| Subramanian & Davey Smith 2006 | 26 states, India,  rural and urban areas | Cross-sectional (subsample of National Family Health Survey from 26 Indian states (1998-99) (n=90,303))  (n=77,220) | Underweight (BMI <16; 16-16.9; 17-18.49; <18.5 kg/m^2^), overweight, obese (BMI 23-24.9; 25-29.9; ≥30 kg/m^2^) | Age, SES, caste, education, living environment, religion, parity, tobacco, alcohol; treatment of morbidities from asthma, malaria, tuberculosis | Not working | 0 | 48,160 |  |  |  |  | 15, 49 | Non-pregnant women not attending school |
|  |  |  |  |  | Non-manual | 0 | 4,433 |  |  |  |  |  |  |
|  |  |  |  |  | Agricultural | 0 | 17,758 |  |  |  |  |  |  |
|  |  |  |  |  | Manual | 0 | 6,869 |  |  |  |  |  |  |
| Wang et al. 2010 | South-western China, Yi farmers from remote mountain areas (Butuo, Zhaojue, Jinyang, Puge, and Xide counties), Yi migrants and Han people from the county seats and Xichang city | Cross-sectional (of the YMS), stratified random cluster sampling (all individuals in clusters sampled)  (n=4,971) | Hypertension (≥130/85 mmHg); overweight and obesity (BMI ≥24 kg/m^2^); smoking | None | Farmer | 675 | 860 |  | 39.2 (12.3) | 40.1 (11.5) |  | ≥20 |  |
|  |  |  |  |  | Migrant | 760 | 546 |  | 40.3 (11.7) | 37.4 (11.7) |  |  |  |
|  |  |  |  |  | Urban | 1,080 | 1,050 |  | 43.6 (13.0) | 45.0 (13.3) |  |  |  |
| Zhou et al. 2003 | Rural areas near big cities in Beijing, Northern China, and Guangzhou, Southern China | Cohort,  sub-sample (<50%) of China-United States Collaborative Study on Cardiovascular and Cardiopulmonary Epidemiology (non-random sample)  (n=633) | Smoking, total cholesterol (millimoles (mmol)/ Litre (l)), triglycerides (mmol/L) | None | Agriculture 1983–84 | 326 | 0 | 326 |  |  |  | 35, 54 (baseline) | Rural areas near big cities |
|  |  |  |  |  | Remained in agriculture 1993–94 |  |  |  |  |  |  |  |  |
|  |  |  |  |  | Agriculture 1983–84 | 102 | 0 | 102 |  |  |  |  |  |
|  |  |  |  |  | Shifted out of agriculture 1993-94 |  |  |  |  |  |  |  |  |
|  |  |  |  |  | Factory work 1983–84 | 135 | 0 | 135 |  |  |  |  |  |
|  |  |  |  |  | Remained in factory work 1993-94 |  |  |  |  |  |  |  |  |
|  |  |  |  |  | Office work 1983–84 | 70 | 0 | 70 |  |  |  |  |  |
|  |  |  |  |  | Remained in office work 1993-94 |  |  |  |  |  |  |  |  |
