# Supplementary material for: Is agricultural engagement associated with lower incidence or prevalence of cardiovascular diseases and cardiovascular disease risk factors? A systematic review of observational studies from low- and middle-income countries
Source: PLoS One. 2020 Mar 31;15(3):e0230744. doi: 10.1371/journal.pone.0230744 (PMC7108743; doi:10.1371/journal.pone.0230744)
Supplement: S4 Table — aThe risk of bias assessment was based on adapted ‘A Cochrane Risk of Bias Assessment Tool: for Non-Randomized Studies of Interventions. (DOCX) [file pone.0230744.s005.docx]

S5 Table Quality of evidence rating^a^ of included studies from five countries (n=13)

| **Author** | **Confounding** | **Selection of participants** | **Measurement of exposure** | **Departures from intended intervention** | **Missing data** | **Measurement of outcome** | **Selection of reported results** | **Overall bias** |
| --- | --- | --- | --- | --- | --- | --- | --- | --- |
| **Addo et al 2006** | Poorly addressed | Poorly addressed | Not described adequately to classify | Not applicable | Well covered | Not described adequately to classify | Poorly addressed | Poorly addressed |
| **Arlappa 2009** | Poorly addressed | Poorly addressed | Not described adequately to classify | Not applicable | Not described adequately to classify | Not described adequately to classify | Poorly addressed | Poorly addressed |
| **Asgary et al 2013** | Poorly addressed | Poorly addressed | Not described adequately to classify | Not applicable | Poorly addressed | Not described adequately to classify | Poorly addressed | Poorly addressed |
| **Balagopal et al 2012** | Poorly addressed | Poorly addressed | Not described adequately to classify | Not applicable | Well covered | Not described | Adequately addressed | Poorly addressed |
| **Gregory et al 2007** | Poorly addressed | Poorly addressed | Not described adequately to classify | Not applicable | Not described adequately to classify | Not described adequately to classify | Poorly addressed | Poorly addressed |
| **Hazarika et al 2004** | Poorly addressed | Poorly addressed | Not described adequately to classify | Not applicable | Not described | Not described adequately to classify | Poorly addressed | Poorly addressed |
| **He et al 1991** | Poorly addressed | Poorly addressed | Not described adequately to classify | Not applicable | Not described | Not described adequately to classify | Poorly addressed | Poorly addressed |
| **Noboro et al 2015** | Poorly addressed | Poorly addressed | Not described adequately to classify | Not applicable | Not described | Not described | Poorly addressed | Poorly addressed |
| **Olugbile & Oyemade 1982** | Poorly addressed | Poorly addressed | Not described adequately to classify | Not applicable | Well covered | Not described | Not described adequately to classify | Poorly addressed |
| **Subasinghe et al 2014** | Poorly addressed | Poorly addressed | Not described adequately to classify | Not applicable | Not described adequately to classify | Not described adequately to classify | Not described adequately to classify | Poorly addressed |
| **Subramanian & Davey Smith 2006** | Poorly addressed | Poorly addressed | Not described adequately to classify | Not applicable | Not described | Not described adequately to classify | Poorly addressed | Poorly addressed |
| **Wang C et al 2010** | Poorly addressed | Poorly addressed | Not described adequately to classify | Not applicable | Not described | Not described | Poorly addressed | Poorly addressed |
| **Zhou et al 2003** | Poorly addressed | Poorly addressed | Not described adequately to classify | Well covered | Not described | Not described adequately to classify | Not described adequately to classify | Poorly addressed |
